# The effects of cognitive-motor multitasking demands on the quality of chest compressions in CPR—A randomized controlled trial

**DOI:** 10.3389/fmed.2025.1536796

**Published:** 2025-02-19

**Authors:** Patricia Hirsch, Kim Pears, Martin Klasen, Christoph Kiefer, Iring Koch, Saša Sopka

**Affiliations:** ^1^Department of Cognitive and Experimental Psychology, Institute of Psychology, RWTH Aachen University, Aachen, Germany; ^2^Interdisciplinary Training Center for Medical Education and Patient Safety—AIXTRA, RWTH Aachen University, Aachen, Germany; ^3^Department of Psychology, Bielefeld University, Bielefeld, Germany; ^4^Department of Anesthesiology, RWTH Aachen University, Aachen, Germany

**Keywords:** resuscitation, cardiopulmonary resuscitation (CPR), chest compression quality, multitasking, task switching

## Abstract

**Background:**

In standard cardiopulmonary resuscitation (CPR), rescuers switch between ventilation and chest compressions. We examined the effect of this task-switching requirement on chest-compression quality to gain insights into the cognitive mechanisms underlying performance in standard CPR. Understanding these mechanisms can help in the development of evidence-based practical implications and cognitive aids for CPR.

**Methods:**

A total of 300 first-year medical and dentistry students (212 females, 20.2 ± 4.4 years) participated in this randomized controlled trial. They received either a CPR training comprising both chest compressions and ventilation (standard CPR) or a CPR training comprising chest compressions only (chest-compression-only CPR). Chest-compression quality (compression depth and rate) was measured via a resuscitation manikin and analyzed using linear mixed models and linear trend analyses.

**Results:**

Overall, chest-compression quality did not differ across standard CPR and chest-compression-only CPR. However, in standard CPR, compression quality was better after ventilation than before ventilation. Importantly, ventilation impaired the quality of the compressions executed immediately after ventilation, but the quality increased with each compression after ventilation, resulting in a better chest-compression quality after ventilation than before it.

**Conclusions:**

This study suggests that ventilation acts as a break, improving physical capability, which in turn enhances compressions after ventilation. However, at the same time, ventilation causes a task switch which increases cognitive demands and impairs chest-compression quality immediately after ventilation. Considering the negative effect of the task-switching demand on chest-compression quality, it is useful to develop cognitive aids for professional medical care. Such cognitive aids can signal an upcoming switch to ventilation, thereby reducing the multitasking load in terms of reduced monitoring demands with respect to the number of chest compressions that have already been executed.

## 1 Introduction

Sudden cardiac arrest is among the leading causes of mortality. Worldwide, 4 to 5 million people die of sudden cardiac arrest every year (1). Cardiopulmonary resuscitation (CPR) is the only lifesaving intervention (2) and the survival rates depend greatly on the quality of CPR (3–7).

The international guidelines published by the American Heart Association (AHA) and the European Resuscitation Council (ERC) (8, 9) recommend CPR that includes both chest compressions and ventilation, also referred to as standard CPR. Laypersons who are unable or unwilling to perform mouth-to-mouth ventilation are recommended to provide at minimum CPR comprising chest-compression-only CPR (10–12). Cco CPR simplifies resuscitation and eliminates concerns about the risk of infection, both of which are aspects that might increase the rate of layperson CPR (12).

A recent study that examined the outcomes after a cardiac arrest in 28 European countries revealed that the survival to hospital rates were greater when laypersons administered standard CPR than when they provided cco CPR (13). However, another study concluded that standard CPR is superior to cco CPR only, when standard CPR is executed correctly (14). Thus, for untrained laypersons, cco CPR may be as effective as standard CPR [see also (15, 16) for reviews].

Regarding the impact of the CPR method on the quality of compressions, there are studies reporting no differences in compression quality (17) and studies that observed better compression quality for standard CPR than for cco CPR or vice versa. For instance, several studies showed that the compression depth and the percentage of compressions with a correct depth were higher for standard CPR than for cco CPR (18, 19). In contrast, in another study, there were more compressions with an adequate depth for cco CPR than for standard CPR (20). This inconsistency might be attributable to differences in the CPR duration and the definition of an adequate depth [e.g., 38 mm in (17) vs. 50–60 mm in (20)].

To analyze time-dependent variations in the compression quality, various studies subdivided the CPR scenario into multiple time intervals (20, 21). These studies showed that in the first minutes, the number of compressions with a correct depth is greater for cco CPR than for standard CPR, but later, it is higher for standard CPR than for cco CPR (19). Moreover, compression quality decreases during both CPR methods (22, 23) and this decrease is greater in cco CPR than in standard CPR (21). This suggests that compressions are physically demanding and can lead to fatigue (24). Consistent with this notion, it has been shown that short breaks of some seconds improve the compression quality in cco CPR, possibly due to reduced rescuer fatigue (25, 26).

So far, differences in compression quality between standard CPR and cco CPR, if existent, are explained by differences in the level of exhaustion caused by these CPR methods. In contrast to these previous studies, in the present study, we focused on cognitive factors that might affect the performance in standard CPR and cco CPR.

From a cognitive perspective, the quality of chest compressions is expected to differ across the CPR methods due to the requirement of switching back and forth between compressions and ventilation. This is because laboratory multitasking studies consistently found that switching between tasks results in performance costs (27–30). More specifically, participants usually need more time to complete a task and commit more errors in switch conditions than in non-switch conditions. These findings suggest that the quality of chest compressions during standard CPR should be worse than that during cco CPR because standard CPR requires rescuers to maintain and update the tasks for compressions and ventilation in working memory, thus potentially inducing general multitasking costs. Chest-compression quality in standard CPR should be further impaired by the requirement to cognitively engage and disengage the chest-compression task when switching between ventilation and compressions. This cognitive demand may result in worse chest-compression quality immediately after ventilation than before ventilation, reflecting switch costs. Thus, multitasking studies suggest that the requirement to switch between ventilation and chest compressions should impair the chest-compression quality in standard CPR.

Despite our improved knowledge of the factors influencing CPR performance [e.g., sex, physical fitness, and body mass index (31–34)], data on the cognitive mechanisms underlying CPR performance is limited. Understanding these mechanisms may help to develop evidence-based practical implications for CPR and cognitive aids for professional medical care. A first step toward that direction is to examine whether the multitasking costs found in laboratory studies also occur in clinical situations.

The established findings from cognitive task-switching research have rarely been considered in resuscitation research. In the present study, we conducted a randomized controlled trial to examine the novel question of whether the switching requirement posed by standard CPR influences chest-compression quality. We hypothesized that chest-compression quality is worse in standard CPR than in cco-CPR (general multitasking costs) and after ventilation than before it (switch costs). To further examine the impact of ventilation on compression quality, we analyzed linear trends in compression quality at a compression-by-compression level (i.e., from one compression to the next compression). Note that previous research was more interested in quantifying changes in compression quality over the entire CPR scenario than in measuring the direct effects of the switch to the ventilation task on compression quality. Therefore, these studies examined the time course of compression quality by comparing the quality across different time intervals. In contrast, in the present study, compression quality was analyzed as a function of the number of compressions that has to be executed before ventilation and the number of compressions that has been performed after ventilation. Thus, the linear trend analysis provides new insights into compression-quality changes before and after ventilation.

## 2 Materials and methods

### 2.1 Selection of participants

The study was conducted during the mandatory introduction weeks at the Medical School RWTH, for which 369 first-year medical and dentistry students were enrolled. The study was approved by the Ethical Committee of the University Hospital—RWTH Aachen (EK328/18), and was conducted in accordance with the Declaration of Helsinki. Written informed consent was obtained from all participants and the study was retrospectively registered with DRKS (DRKS00027102). Note that since this is an educational study, there is no need for preregistration on a clinical registry in accordance with national academic principles.

### 2.2 Study design and setting

We designed the study as a randomized controlled trial and evaluated participants' basic life support (BLS) skills in a parallel-group design with two study arms. CPR performance was analyzed after BLS training in two separate conditions.

BLS training covered 2 days, with 1 week in between. On Day 1, participants were trained in a single-rescuer CPR algorithm followed by a two-rescuer algorithm on Day 2. BLS training was conducted in groups of 10 to 15 participants each and was guided by professional instructors using Peyton's four-step-approach, which is widely used in BLS training (35).

### 2.3 Interventions

**Figure 1:**
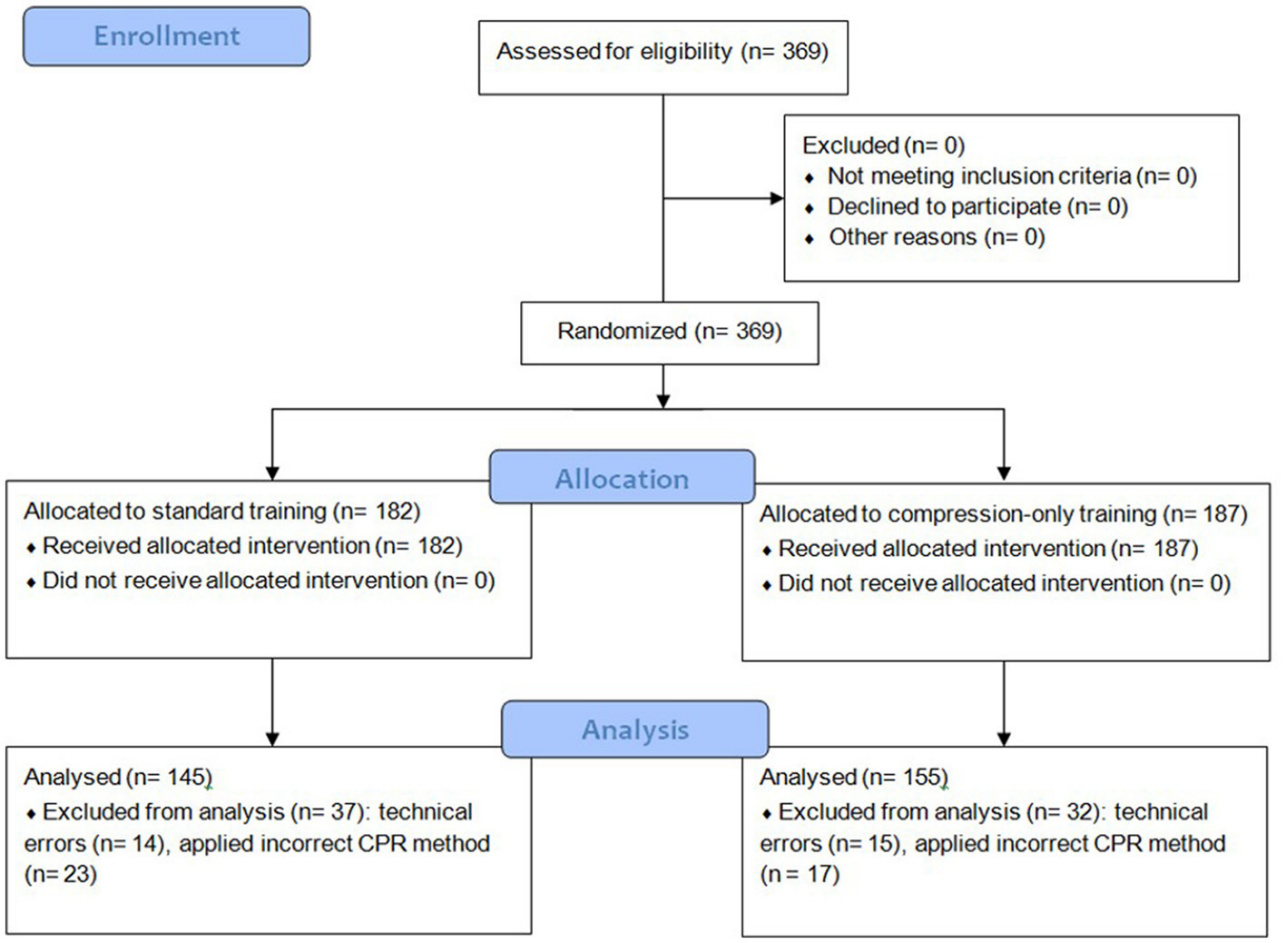
Consort flow chart.

There were two study arms to which participants were randomly assigned (Figure 1).

#### 2.3.1 Study arm 1: standard CPR training condition

The first training session was based on current ERC guidelines, which recommend chest compressions and ventilation. During the second training, the participants learned the two-rescuer CPR algorithm. This algorithm included the application of an automated external defibrillator (i.e., AED) and a change of helpers. This study arm represents the mandatory BLS training for medicine students at RWTH Aachen University.

#### 2.3.2 Study arm 2: cco CPR training condition

The participants first learned a single-rescuer CPR algorithm including chest compressions only. During the second training, in addition to the two-rescuer CPR algorithm of the first study arm, they learned the standard single-rescuer CPR algorithm including ventilation to ensure that all participants could conduct rescue operations in accordance with the ERC guidelines and the mandatory BLS training at RWTH Aachen University.

### 2.4 Measurements and outcomes

CPR performance was measured at three time points: the baseline test immediately before the first training, the post-test directly after the first training on Day 1 (i.e., after the standard CPR or cco CPR training), and the follow-up test 6 months after the second training. A standardized mock cardiac arrest single-rescuer scenario was used to assess the BLS performance. The participants were asked to resuscitate a collapsed person represented by a manikin (Resusci Anne^TM^, Laerdal) by performing all the steps they would take in a real-life emergency situation until a stop signal was given (120 s after the first compression).

In contrast to the baseline test, CPR performance was measured twice during both the post-test and the follow-up test. Independently of the training condition, participants performed CPR once with ventilation and once without ventilation with a break of 30 s in between. Whether the performance assessment started with the standard or the cco-CPR was counterbalanced across participants. CPR performance comprising compression depth, percentage of compressions with a correct depth, compression rate, and percentage of compressions with a correct rate was measured via the manikin with a computer-based software (Laerdal PC SkillReporting System Software, Version 2.4.1).

In addition, demographic data were recorded via an online questionnaire before the baseline test. We also employed online questionnaires before the baseline test and after the training day 1 and the training day 2 to record knowledge of CPR, self-related confidence in providing CPR, and training evaluation.

### 2.5 Sample size planning

We conducted an a-priori sample size calculation with G^*^Power (Version 3.1.9.6) and set the α level at 0.05 and the power (1-β) at 95%. A priori effect sizes (d) for general multitasking costs were estimated based on previous studies (36–41) which reported sufficient information to allow for effect size (d) calculation.

Since most of these studies had small sample sizes, effect sizes varied substantially, with smaller samples tending toward larger effects (explorative correlations r = −0.47 for the studies reporting compression depth and r = −0.59 for the studies reporting compression rate). This is consistent with previous reports that studies with small sample sizes tend to overestimate effect sizes (42). Based on the aforementioned studies, average effect sizes were 0.955 for compression depth and 1.668 for compression rate. Since sample sizes and effect sizes varied greatly among the studies, we decided to settle conservatively for a medium effect size of 0.5 for compression depth and a large effect size of 0.8 for compression rate. This resulted in recommended sample sizes of *n* = 210 for the compression depth and *n* = 84 for the compression rate. Both were adequately covered by the maximum available sample of 369 students enrolled.

For switch costs, we did not conduct an a-priori sample size calculation because there were no studies assessing this performance decline in CPR.

### 2.6 Randomization

An independent employee, who was blinded for the scientific investigation, randomized and allocated the students into groups of 10 to 15 persons. Each day, we trained six groups, three in each study arm. Allocation of the groups to the study arms was performed following a balanced sequence of randomly generated numbers.

### 2.7 Statistical analysis

We examined the effects of multitasking demands posed by standard CPR on chest-compression quality by means of generalized mixed models and linear trend analyses. In all the analyses, we employed the CPR performance after the first training (post-test). We used linear mixed models to account for the nesting of compressions within persons.

The analyses were conducted using the R packages lme4 (43) and lmerTest (44). For the continuous outcomes (compression depth in mm and compression rate in compressions per minute, cpm), a linear mixed model was estimated with a restricted maximum likelihood (REML) procedure. The significance testing of regression coefficients was done via *t*-tests using Satterthwaite's approximation for degrees of freedom (45). The reported effect sizes for the linear mixed models are computed according to Hedges (46) and can be interpreted like Cohen's *d*. For the binary outcomes (correct compression depth and rate: yes/no), a logistic mixed model was estimated with maximum likelihood estimation. The significance testing of regression coefficients was done via Wald tests. The reported effect sizes for the logistic mixed models are standardized average marginal effects.

The analyses focused on two contrasts. First, general multitasking costs were assessed by analyzing the independent between-subjects variable CPR method (cco CPR vs. standard CPR). For this analysis, we used the cco CPR performance of the cco training group and the standard CPR performance of the standard training group. This approach is similar to that reported in previous studies which averaged compression quality across the entire CPR duration and contrasted it across the CPR methods (20). For this analysis, we used all chest compression because the working memory demands (i.e., to maintain the compression and ventilation tasks in standard CPR vs. compression task in cco CPR) were constant across the entire CPR duration and should, therefore, affect each single chest compression.

Second, switch costs were assessed based on the within-subjects independent variable time point of the chest compression (before vs. after ventilation). This analysis was restricted to the standard CPR performance in the standard training group. We compared performance in the last five compressions before ventilation (task repetition) with that in the first five compressions after ventilation (task switches).^1^ Note that the effect of a task switch can only be observed immediately after the switch (27). Hence, from an empirical perspective, it is required to focus on the first compressions following the switch, to isolate switch costs. Moreover, we conducted linear trend analyses to explore linear trends in the quality of the last five chest compressions before ventilation and the first five compressions after ventilation.

The dependent variable was chest-compression quality, defined according to current guidelines. We analyzed chest-compression depth (recommended: 50–60 mm), percentage of compressions with a correct depth, compression rate (recommended: 100–120 cpm), and percentage of compressions with a correct rate.^2^

## 3 Results

### 3.1 Sample characteristics

Of the 369 students enrolled in the course, 69 students had to be excluded from the study due to missing data and technical problems (Figure 1). Our final data set included 300 participants for the general multitasking cost analysis and 145 participants for the switch cost analysis and linear trend analysis (see Table 1 for demographics).

**Table 1:** Demographic data of the final sample.

	**General multitasking costs analysis**	**Switch costs analysis**
N	300	145
Sex	212 female 74 male 14 without specification	102 female 34 male 9 without specification
Age	20.2 ± 4.4 years	20.1 ± 4.5 years
With previous CPR training experience	52	29

### 3.2 Mixed models on general multitasking costs

**Figure 2:**
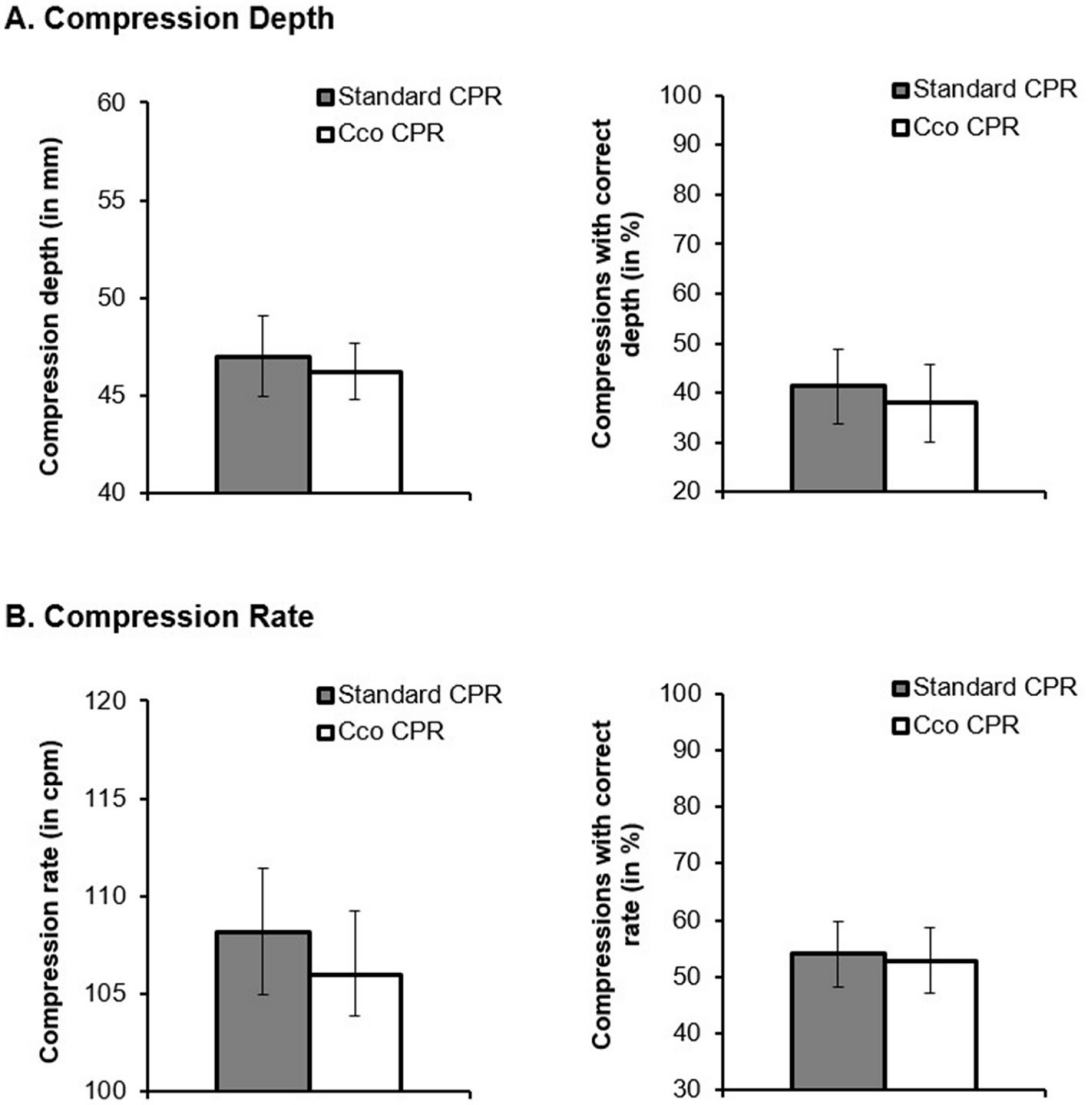
**(A)** Compression depth (in ms) and compressions with a correct depth (in %) and **(B)** compression rate (in cpm) and compressions with a correct rate (in %) as a function of the CPR method (standard CPR vs. chest-compression-only CPR; cco CPR).

Regarding chest-compression depth in the post-test, we did not find significant differences across cco CPR and standard CPR. Neither compression depth, *t*_(299.006)_ = 0.719, *p* = 0.473, *d* = 0.207, CI = [−0.357, 0.772], nor percentage of compressions with a correct depth on the log odds ratio scale, *z* = 0.751, *p* = 0.453, average marginal effect = 0.034, *d* = 0.089, CI = [−0.143, 0.320], differed significantly across the CPR methods (Figure 2A).

Like chest-compression depth, compression rate, *t*_(298.961)_ = 1.321, *p* = 0.188, *d* = 0.480, CI = [−0.232, 1.192], and percentage of chest-compressions with a correct rate, *z* = 0.289, *p* = 0.772, average marginal effect = 0.012, *d* = 0.032, CI = [−0.186, 0.250], also did not differ significantly across cco CPR and standard CPR (Figure 2B).

### 3.3 Mixed models on switch costs

**Figure 3:**
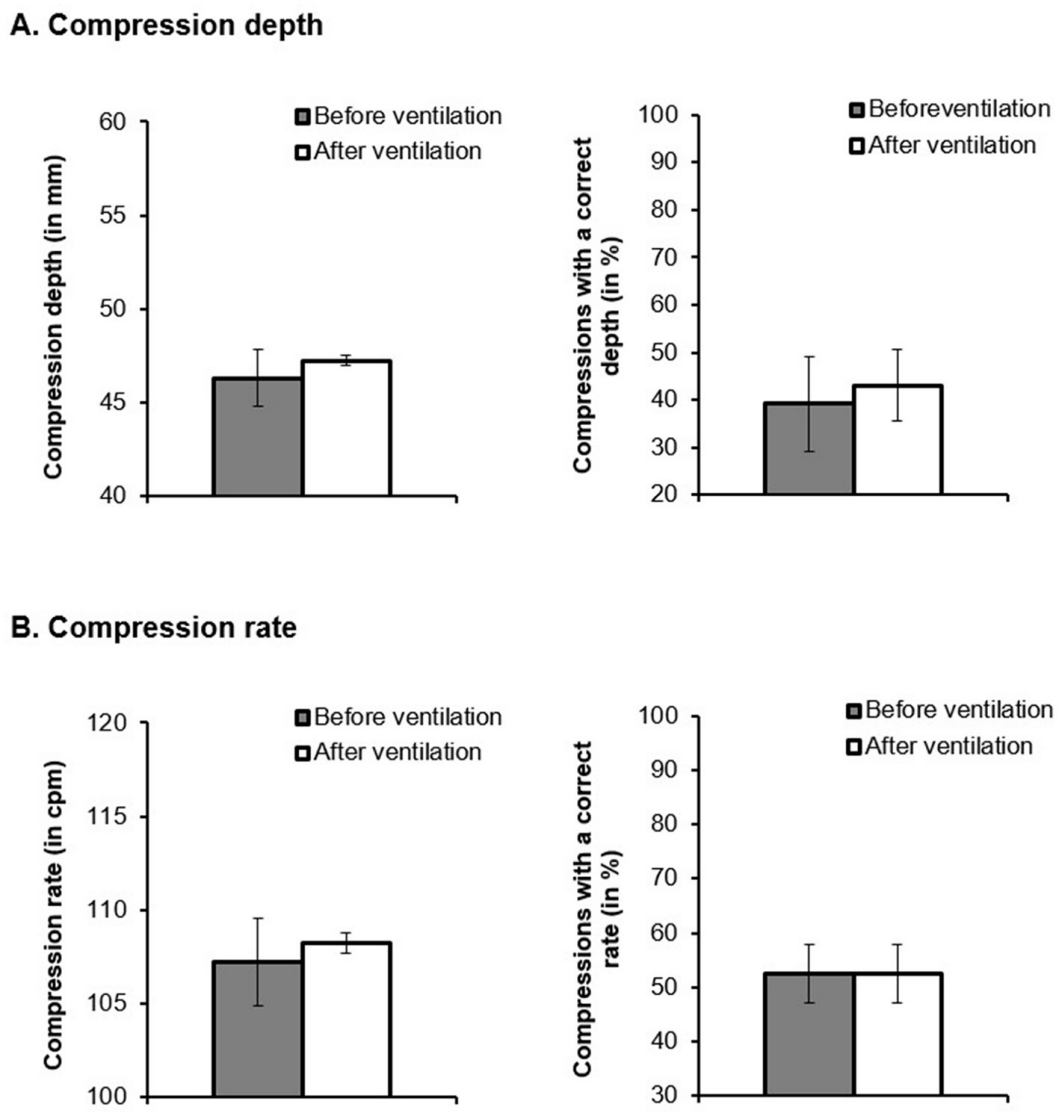
**(A)** Compression depth (in ms) and compressions with a correct depth (in %) and **(B)** compression rate (in cpm) and compressions with a correct rate (in %) as a function of the time point of chest compressions (before vs. after ventilation).

The chest-compression depth in the post-test was significantly deeper after than before ventilation, *t*_(4513.297)_ = 6.644, *p* < 0.001, *d* = 0.246, CI = [0.137, 0.318] (Figure 3A). Moreover, the percentage of chest-compressions with a correct depth was significantly higher after ventilation than before it, *z* = 4.862, *p* < 0.001, average marginal effect = 0.0395, *d* = 0.097, CI = [0.058, 0.136].

The compression rate was also significantly higher after ventilation than before it, *t*_(3563.806)_ = 3.533, *p* < 0.001, *d* = 0.212, CI = [0.094, 0.33]. The percentage of chest-compressions with an adequate rate did not change significantly after ventilation, *z* = 0.0798, *p* = 0.936, average marginal effect = 0.00103, *d* = 0.003, CI = [−0.079, 0.086] (Figure 3B).

### 3.4 Linear trend analysis

**Figure 4:**
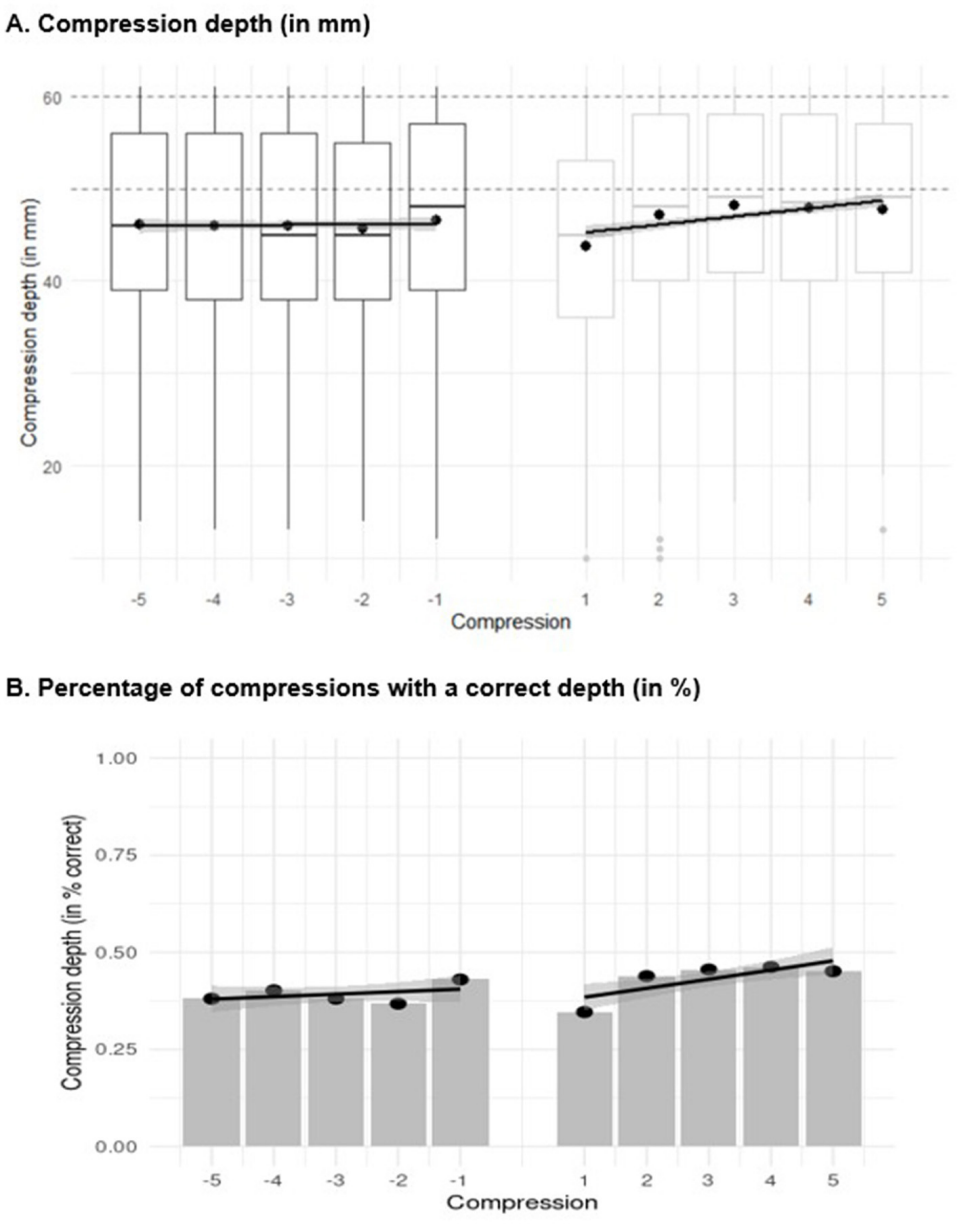
Linear trend analysis for **(A)** compression depth and **(B)** the percentage of chest compressions with a correct depth of the last five compressions before ventilation (i.e., −5 to −1) and the first five compressions after ventilation (i.e., 1 to 5). In figure **(A)**, the boxplots show the median (horizontal line), the 1^st^ and 3^rd^ quartiles (box), and a maximum of 1.5 times the interquartile range outside the box (whiskers). Values that are further outside the box are displayed as individual points. In the middle of the box, the mean is marked with an additional point. In figure **(B)**, the points depict the percentage of chest compression with a correct depth and the regression lines represent the predicted values due to the linear trend.

For compression depth, there was no significant trend in the five last chest compressions before the ventilation (*b* = 0.0635), *t*_(4511.286)_ = 0.933, *p* = 0.351, meaning that compression depth did not change before ventilation. Immediately after ventilation, the compression depth decreased significantly (−0.939), *t*_(4511.286)_ = −3.980, *p* < 0.001, but the slope increased significantly (0.803), *t*_(4511.286)_ = 8.336, *p* < 0.001. Thus, the first compression after a preceding shift to ventilation was shallower than the last compression before ventilation, but the depth increased with each compression after ventilation (Figure 4A).

Regarding the percentage of compressions with a correct depth, on the log odds ratio scale, there was a significant trend in the five last compressions before ventilation (*b* = 0.0913), *z* = 269.030, *p* < 0.001, indicating that the percentage of compressions with a correct depth increased significantly with each compression before ventilation (Figure 4B). After ventilation, the percentage of compressions with a correct depth decreased significantly (−0.298), *z* = −877.741, *p* < 0.001, meaning that the percentage of compressions with a correct depth was lower for the first compression after ventilation than for the last compression before ventilation. However, the slope of compressions with a correct depth increased significantly after ventilation (0.238), *z* = 700.985, *p* < 0.001.

**Figure 5:**
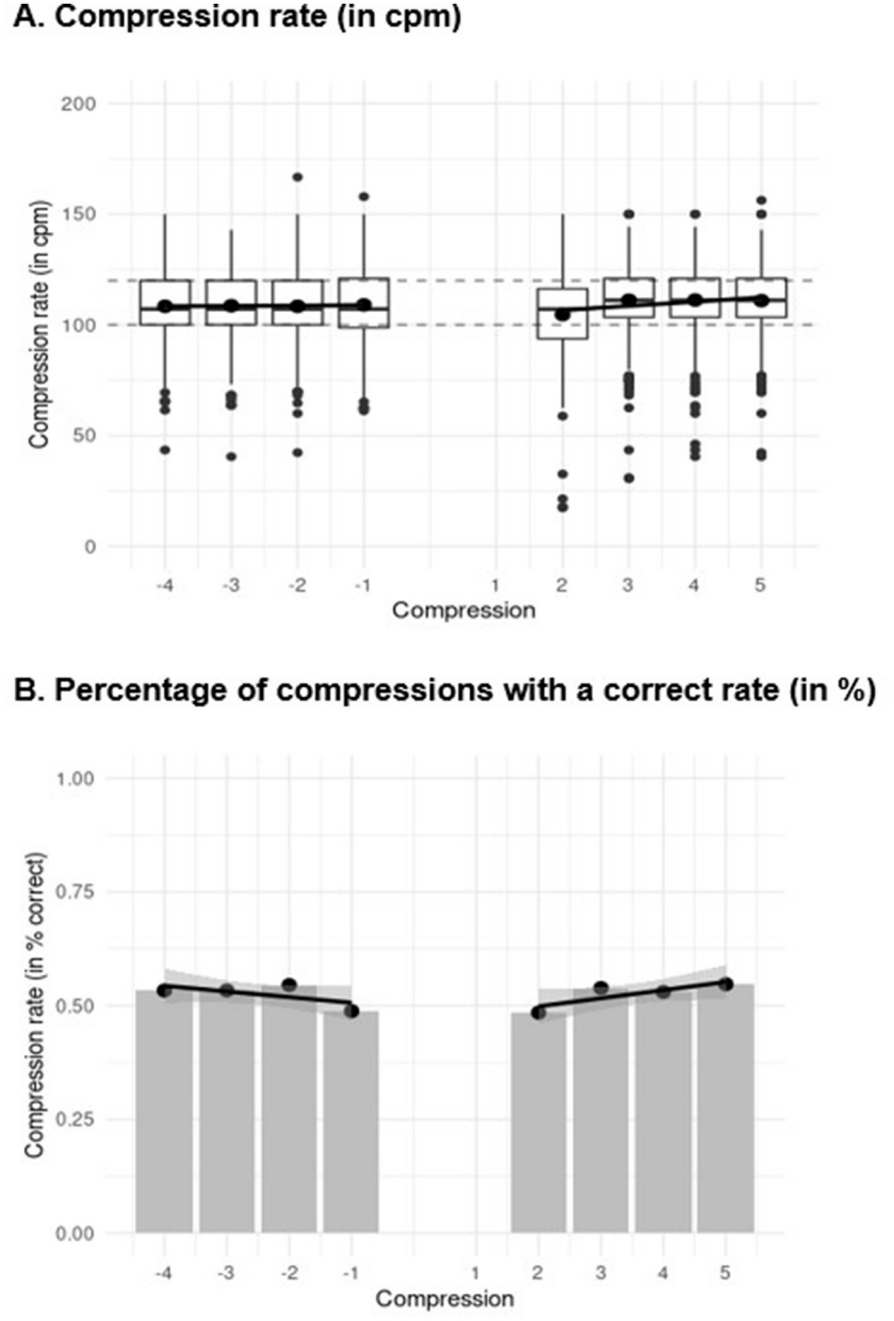
Linear trend analysis for **(A)** chest compression rate and **(B)** the percentage of chest compressions with a correct rate of the five compressions before ventilation (i.e., −4 to −1) and the first compressions after ventilation (i.e., 2 to 5). In figure **(A)**, the boxplots show the median (horizontal line), the 1^st^ and 3^rd^ quartiles (box), and a maximum of 1.5 times the interquartile range outside the box (whiskers). Values that are further outside the box are displayed as individual points. In the middle of the box, the mean is marked with an additional point. In figure **(B)**, the points depict the percentage of chest compression with a correct rate and the regression lines represent the predicted values due to the linear trend.

For compression rate, there was no significant trend before ventilation (*b* = 0.135), *t*_(3561.807)_ = 0.933, *p* = 0.453, indicating that compression rate did not change before ventilation (Figure 5A). However, after ventilation, the compression rate significantly decreased (−3.794), *t*_(3561.811)_ = −6.359, *p* < 0.001, and the slope of the rate increased significantly (1.706), *t*_(3561.811)_ = 6.712, *p* < 0.001. Thus, the first compression rate after ventilation was lower than the last rate before it and the rate increased with each compression after ventilation.

Regarding percentage of compressions with a correct rate, on the log odds ratio scale, there was no significant trend before ventilation, (*b* = −0.084), *z* = −1.596, *p* = 0.111. Thus, the percentage of compressions with a correct rate did not change across the last five compressions before ventilation (Figure 5B). After ventilation, the percentage of compressions with a correct rate decreased significantly, (−0.171), *z* = −0.982, *p* = 0.326, and also the slope of the percentage of compressions with an adequate rate increased significantly (0.206), *z* = 2.757, *p* = 0.006, indicating that the first compression rate after ventilation was less often in the recommended range than the last compression before ventilation. However, the percentage of compressions with a correct rate increased with each compression after ventilation.

## 4 Discussion

The aim of the present study was to examine the impact of multitasking demands on CPR quality. With respect to general multitasking costs we found that despite the requirement to interrupt chest compressions and switch to the ventilation task before switching back to chest compressions, the compression quality did not differ across cco CPR and standard CPR. However, during standard CPR, the compression depth, the number of chest compressions with a recommended depth, and the compression rate were higher after ventilation than before it. From the cognitive perspective, task switching was assumed to impair the quality of chest compressions in standard CPR compared to cco CPR and to result in worse chest compression quality after ventilation than before ventilation (21, 28, 47). Importantly, in line with the cognitive perspective, the first compression after ventilation had a lower rate, was less deep and more often incorrect in its depth and rate than the last compression before ventilation, but the depth, the rate, and the percentage of compressions with recommended depth and rate increased with each ventilation after ventilation. This increase resulted in a better compression quality after than before ventilation.

### 4.1 The role of cognitive multitasking in CPR

A reason for the comparable performance across the CPR methods might be that the performance costs in standard CPR, which were expected from the cognitive perspective (27–30), were overshadowed by motor performance benefits caused by the break. CPR is a task encompassing both a cognitive and a motor component. This is because, in addition to the maintenance and updating of the compression and ventilation tasks as well as the switching between tasks, rescuers are required to carry out potentially fatiguing movements in terms of chest compressions which require sufficient force to be executed. Evidence for the notion that CPR is physically demanding is provided by various studies which showed that during CPR, the quality of chest compressions declines rapidly over time (20, 22, 23).

Ventilation could possibly act as a temporal break that increases the physical capabilities during CPR delivery (40, 48). Accordingly, previous studies observed that the heart rate of rescuers increases more during cco CPR than during standard CPR, whereas the chest-compression quality decreases more rapidly during cco CPR than during standard CPR (19, 20). This suggests that standard CPR rescuers may have more force to conduct compressions, especially after ventilation, and that the performance cost due to the requirement of maintaining and updating multiple tasks in working memory might be overshadowed. Correspondingly, we found that the chest-compression depth was deeper after ventilation than before it.

In fact, Heidenreich and colleagues (2006) (20) observed that cco CPR resulted in more adequate compressions than standard CPR for the first 2 min of CPR. After 3 min, the difference in the number of adequate compressions across the CPR methods, however, diminished or even reversed into more adequate chest compressions with standard CPR than cco CPR (19, 20). These findings suggest that when physical exertion is less pronounced, the cognitive demands of maintaining and updating the ventilation and chest compression tasks in working memory as well as switching between these tasks may impair chest-compression performance.

Note that the depth of the first compression after ventilation was lower than the last compression executed before ventilation, but the compression depth then increased rapidly with each compression. A similar data pattern was observed for the compression rate. This novel finding of a decline in the depth and rate immediately after ventilation might reflect performance costs caused by the switching requirement in standard CPR and thus be of cognitive nature. In addition, the novel finding of a rapid increase in the depth and rate in the following chest compressions might reflect the motor-related benefit caused by the ventilation break and thus might have a motor-related nature. Importantly, previous studies typically averaged the compression quality across specific time intervals and were, therefore, not able to isolate these effects.

### 4.2 Limitations

A limitation of this study is that—despite the large sample size—our sample does not represent the general public regarding age, fitness, and education. Our sample consisted of young highly-educated medical and dentistry students. Thus, they do not necessarily represent the average person who would perform CPR in a real-life situation.

Furthermore, CPR on a manikin does not perfectly mimic clinical CPR. The simulation conditions may also help participants feel less emotional arousal or be less committed to resuscitation than in a real situation.

Moreover, we focused on the compression depth and compression rate as indices of CPR quality. We thus cannot draw conclusions about the impact of multitasking demands on the quality of the overall CPR performance. To do so, a single measure representing the overall CPR performance is required.

### 4.3 Summary and conclusion

By transferring concepts from cognitive psychology (27–30) to resuscitation research, we gained novel knowledge about the effects of ventilation on chest-compression performance in CPR. The present study suggests that ventilations act as a break, improving physical capability, which in turn enhances compressions after ventilation. At the same time, ventilation are associated with a task switch which hampers compression quality immediately after ventilation. Thus, the effects of ventilation on chest-compression performance rely on motor-related and cognitive mechanisms. If we consider the evidence for negative effects of task switching in the standard CPR method, it may be useful to develop cognitive aids for professional medical care. Such cognitive aids can act as a task cue for an impending switch to ventilation. This could reduce the mental multitasking load because there would be reduced monitoring demands with respect to the number of chest compressions already performed.

## Data Availability

The raw data supporting the conclusions of this article will be made available by the authors, without undue reservation.

## Footnotes

^1^We replicated the analyses with 8, 10, and 15 compressions before and after the ventilation. The results were identical for each number of compressions. However, with 15 compressions, the effect sizes were smaller.

^2^Chest-compression depth is measurable for each compression, whereas chest-compression rate is calculated as a difference measure between two compressions. Therefore, the analysis of compression depth is based on five means before the ventilation and five means after the ventilation (i.e., one mean for each compression), whereas four means are used for the analysis of compression rate. Importantly, the means are calculated for both compression depth and rate using the same compressions.
